# Sociodemographic predictors of multiple non-communicable disease risk factors among older adults in South Africa

**DOI:** 10.3402/gha.v6i0.20680

**Published:** 2013-09-16

**Authors:** Nancy Phaswana-Mafuya, Karl Peltzer, Witness Chirinda, Alfred Musekiwa, Zamakayise Kose

**Affiliations:** 1 HIV/AIDS/STI/and TB (HAST), Human Sciences Research Council, Pretoria, Port Elizabeth, South Africa; 2 Office of the Deputy Vice Chancellor, Nelson Mandela Metropolitan University, Port Elizabeth, South Africa; 3 Department of Psychology, University of Limpopo, Turfloop Campus, Sovenga, South Africa; 4 ASEAN Institute for Health Development, Mahidol University, Salaya, Thailand; 5 Faculty of Health Sciences, Wits Reproductive Health and HIV Institute, University of the Witwatersrand, Johannesburg, South Africa

**Keywords:** self-reported, risk factors, ageing, South Africa

## Abstract

**Background and objective:**

Unhealthy lifestyle behaviours are important risk factors of morbidity and mortality. This study aimed to explore the sociodemographic predictors of multiple non-communicable disease (NCD) risk factors experienced by elderly South Africans.

**Methods:**

We conducted a national population-based cross-sectional survey with a sample of 3,840 individuals aged 50 years or above in South Africa in 2008. The outcome variable was the co-existence of multiple NCD risk factors (tobacco use, alcohol, physical inactivity, fruit and vegetable intake, overweight or obesity, and hypertension) in each individual. The exposure variables were sociodemographic characteristics, namely, age, gender, education, wealth status, population group, marital status, and residence. Multivariate linear regression was used to assess the association between sociodemographic variables and multiple NCD risk factors.

**Results:**

The mean number of NCD risk factors among all participants was three (95% confidence interval: 2.81–3.10). Multivariate linear regression analysis revealed that being female, being in the age group of 60–69 years, and being from the Coloured and Black African race were associated with a higher number of NCD risk factors. Marital status, educational level, wealth, and residence were not significantly associated with the number of NCD risk factors experienced.

**Conclusions:**

The co-existence of multiple lifestyle NCD risk factors among the elderly is a public health concern. Comprehensive health-promotion interventions addressing the co-existence of multiple NCD risk factors tailored for specific sociodemographic groups are needed.

There are well-documented key risk factors for non-communicable diseases (NCDs). These risk factors include unhealthy lifestyle behaviours such as high tobacco and alcohol consumption, an unhealthy diet, physical inactivity, and raised blood pressure. They define the occurrence and severity of NCDs (1, 2) such as cancers and cardiovascular diseases which generally develop from the interaction of multiple risk factors. There is an increase in NCD risk factors in South Africa (3, 4), including among elderly South Africans (3), which may place a heavy burden on the already constrained healthcare system (3, 5).

Limited studies have been conducted to determine the association between sociodemographic characteristics and multiple NCD risk factors among the elderly in developing countries. Most previous studies have concentrated on the significance of one unhealthy behaviour in an individual and focused less on other unhealthy behaviours that may coexist within an individual. In a cross-sectional study in three rural sites in Malawi, Rwanda, and Tanzania, results from five risk factors that were examined (alcohol intake, smoking, vigorous physical activity, hypertension, and overweight) showed that individuals aged 50 years and above were more likely to have multiple risk factors (6). Among men aged 50 years and above, 49.5% had two or more risk factors in comparison to 52.0% of women of the same age. Alcohol intake among men aged 50–59 years was reported to be at its peak (i.e. women are reported to have increased alcohol intake with increased age). In a study conducted by Minh et al. (7) in Vietnam, hypertension was directly associated with socioeconomic status among men. Inequalities in health between socioeconomic groups and the contribution of lifestyle factors to these inequalities have been evident from other international studies as well (8, 9). In order to reduce the burden of NCDs, healthy lifestyles need to be promoted from an early age as prevention is better than cure.

While the association between sociodemographic characteristics and multiple NCD risk factors has been reported elsewhere, limited studies, if any, have reported this among elderly South Africans (10, 11). Research identifying the association between sociodemographic characteristics and multiple NCD risk factors among elderly South Africans is imperative given the urgent need to address health disparities. Against this background, this study explores the association between sociodemographic characteristics and multiple NCD risk factors among elderly South Africans who participated in the Study of Global Ageing and Adult Health (SAGE wave 1) in 2008.

## Methodology

### Description of survey and study population

We conducted a national population-based cross-sectional survey with a sample of 3,840 aged 50 years or above in South Africa in 2008. The SAGE sample design entails a two-stage probability sample that yields national estimates to an acceptable precision at the provincial level, by locality type (urban and rural), and by race (Black Africans, Coloured/mixed race, Indian or Asian, and White). The individual response rate among those aged 50 years or older was 77%. Further sampling details have been published elsewhere (12). SAGE wave 1 was carried out in South Africa by the Human Sciences Research Council (HSRC) in partnership with the World Health Organization (WHO) and the National Department of Health (NDOH). The study was approved by the HSRC Research Ethics Committee (REC 5/13/04/06) and the NDOH (J1/14/45).

### Measures

The outcome variable was the number of multiple NCD risk factors (tobacco use, alcohol, physical inactivity, fruit and vegetable intake, overweight or obesity, and hypertension) in each individual. The exposure variables were sociodemographic characteristics, namely, age, gender, education, wealth status, race, marital status, and residence.

#### Blood pressure (systolic and diastolic)

Blood pressure (systolic and diastolic) was measured three times on the right wrist of the seated respondent using an automated recording device (OMRON R6 Wrist Blood Pressure Monitor, HEM-6000-E, Omron Healthcare Europe, Hoofddorp, the Netherlands). Out of three measurements, the average of the last two readings was used, as suggested by WHO (13). In accordance with the Seventh Report of the Joint National Committee of Prevention, Detection, Evaluation, and Treatment of High Blood Pressure, individuals with systolic blood pressure ≥140 mm Hg and/or diastolic blood pressure ≥ 90 mm Hg and/or who reported the current use of anti-hypertensive medication were considered to be suffering from high blood pressure (14).

#### Tobacco use

Lifetime tobacco use was assessed with the question ‘Have you ever smoked tobacco or used smokeless tobacco?’ Lifetime tobacco users were asked, ‘Do you currently use (smoke, sniff, or chew) any tobacco products such as cigarettes, cigars, pipes, chewing tobacco, or snuff?’ The response options were ‘Yes, daily’, ‘Yes, but not daily’, and ‘No, not at all’. These questions were derived from the WHO Guidelines for Controlling and Monitoring the Tobacco Epidemic (15). Participants who responded with ‘Yes, daily’ were classified as daily tobacco users.

#### Alcohol use

Lifetime alcohol use was assessed with the question ‘Have you ever consumed a drink that contains alcohol (such as beer, wine, spirits, etc.)?’ Response options were ‘Yes’ or ‘No, never’. Lifetime alcohol users were asked about current (past month) alcohol use, and current alcohol users were asked, ‘During the past 7 days, how many drinks of any alcoholic beverage did you have each day?’ Risky drinking was defined in two ways: heavy drinkers (>7 drinks/week) and binge drinkers (>3 drinks/one occasion/week). These are considered ‘risky drinking’, according to the National Institute on Alcohol Abuse and Alcoholism (NIAAA) (16).

#### Fruit and vegetable consumption

Fruit and vegetable consumption was assessed using two questions: ‘How many servings of fruit do you eat on a typical day?’ and ‘How many servings of vegetables do you eat on a typical day?’ using 24 h dietary recall data as the gold standard (17). Researchers were trained to standardize the serving size and number of servings reported. Insufficient fruit and vegetable consumption was defined as fewer than five servings of fruits and/or vegetables per day (17). Cronbach's α for the two questions in this sample was 0.74.

#### Height and weight

Height and weight were measured using a stadiometer and a calibrated weighing scale, respectively. Body Mass Index (BMI) was used as an indicator of obesity (≥30 kg/m^2^). BMI was calculated as weight in kilograms divided by height in metres squared. Overweight was defined as having a BMI ≥ 25, and obesity as BMI ≥ 30.

#### Physical activity

Physical activity was measured using the General Physical Activity Questionnaire (GPAQ). The instrument gathered information on physical activity in three domains (activity at work, travel to and from places, and recreational activities), as well as time spent sitting. The questionnaire also assessed vigorous and moderate activities performed at work and for recreational activities.

Information on the number of days per week spent on different activities and the time spent in a typical day for each activity was also recorded (18). For physical activity, in addition to the total minutes of activity, the activity volume was also computed by weighing each type of activity by its energy requirement in metabolic equivalents (METs). One MET was defined as the energy cost of sitting quietly and was equivalent to a caloric consumption of 1 kcal/kg/hour. A MET-minute showed the total activity volume on a weekly basis, and it was calculated by multiplying time spent on each activity during a week by the MET values of each level of activity. MET values for different level activities were set as 4 MET for moderate-intensity physical activity, 8 MET for vigorous physical activity, and 4 MET for transport-related walking or cycling. The total physical activity for GPAQ was calculated as the sum of total moderate, vigorous, and transport-related activities per week. The number of days and total physical activity in MET-minutes per week were used to classify respondents into three categories of physical activity: a low, moderate, or high level. A person reaching any of the following criteria is classified in the ‘high physical activity’ category: vigorous intensity activity for at least 3 days per week, achieving a minimum of at least 1,500 MET-minutes per week; or 7 or more days of any combination of walking and moderate vigorous intensity activities per week, achieving a minimum of at least 3,000 MET-minutes per week. A person who is not meeting the criteria for the ‘high’ category but is meeting any of the following criteria is classified in the ‘moderate physical activity category’: 3 or more days per week of vigorous intensity activity for at least 20 minutes per day, 5 or more days per week of moderate-intensity activity or walking for at least 30 minutes per day, or 5 or more days per week of any combination of walking and moderate- or vigorous intensity activities, achieving a minimum of at least 600 MET-minutes per week. A person not meeting any of the above-mentioned criteria falls in the **‘**low physical activity’ category. Physical inactivity was defined as those who had low levels of physical activity; moderate and high levels of physical activity were collapsed in further analysis (18).

#### Economic or wealth status

It is not easy to obtain accurate income data from household surveys, yet these are crucial because of the relationship between health and wealth. We therefore used household assets and characteristics of the dwelling, retirement and retirement benefits, financial security, income, consumption, and financial transfers as income estimates. A random-effects probit model was used to identify indicator-specific thresholds that represent the point on the wealth scale above which a household is more likely than not to own a particular asset. This enabled an estimation of an asset ladder using a Bayesian post-estimation (empirical Bayes) method. These estimates of thresholds, combined with actual assets observed to be owned for any given household, were used to produce an estimate of household-level wealth status. This was used to create wealth quintiles (19). The wealth quintiles were collapsed in the analysis to tertiles (1st and 2nd quintiles: low; 3rd quintile: medium; and 4th and 5th quintiles: high).

### Data analysis

The data were entered into CSPro and analysed using STATA Version 10. Data were weighted using post-stratified individual probability weights based on the selection probability at each stage of selection. Individual weights were post-stratified by province, sex, and age groups according to the 2009 Medium Mid Year population estimates from Statistics South Africa, which are available at http://www.statssa.gov.za/publications/P0302/P03022009.pdf. Multivariate linear regression was used to assess the effect of sociodemographic variables on frequency of NCD risk factors. Non-collinear variables statistically significant at the *p*<0.05 level in bivariate analyses were included in the multivariate model. In the analysis, weighted percentages are reported.

## Results

### Sample characteristics

The total sample included 3,840 older South Africans aged 50 years and above; 44.1% were men and 55.9% were women. The most prevalent population group was Black Africans (74%), and almost half of the participants (49.9%) were aged between 50 and 59 years. The educational level of most participants (71.6%) was lower than secondary school education, and almost two-thirds (64.9%) lived in an urban area (Table 1).

**Table 1:** Sociodemographic sample characteristics of older South Africans

Variables	Total sample	Men	Women
	*N* (%)	*N* (%)	*N* (%)
All	3,840 (100)	1,636 (44.1)	2,204 (55.9)
Age			
50–59	1,695 (49.9)	757 (52.1)	938 (48.1)
60–69	1,233 (30.6)	537 (30.7)	696 (30.6)
70 and over	912 (19.5)	344 (17.2)	568 (21.3)
Race			
African Black	2,053 (74.0)	803 (73.8)	1,250 (74.2)
White	269 (9.3)	132 (10.7)	137 (8.3)
Coloured	655 (12.8)	232 (11.8)	423 (13.6)
Indian or Asian	307 (3.8)	136 (3.7)	171 (3.9)
Marital status			
Single	512 (14.3)	142 (8.5)	370 (19.0)
Married or cohabiting	2,007 (55.9)	1,230 (80.0)	777 (36.6)
Separated or divorced	230 (5.9)	87 (3.8)	143 (7.6)
Widow	1,020 (23.9)	159 (7.7)	861 (36.8)
Educational level			
No schooling	645 (25.5)	371 (33.9)	274 (18.2)
Less than 7 years	874 (27.0)	340 (25.4)	534 (28.4)
8–11 years	1,068 (32.7)	417 (26.1)	651 (38.4)
12 or more years	399 (14.9)	195 (14.7)	204 (15.0)
Wealth status			
Low	1,482 (40.6)	621 (40.6)	39.3 (40.6)
Medium	731 (18.2)	259 (13.2)	21.5 (22.2)
High	1,608 (41.2)	748 (46.2)	39.2 (37.2)
Geolocality			
Rural	1,276 (35.1)	561 (34.1)	715 (35.9)
Urban	2,561 (64.9)	1,076 (65.9)	1,485 (64.1)

### NCD risk factors

The distribution of the six NCD risk factors by sociodemographic variables is shown in Table 2. The overall prevalence of daily tobacco consumption was 19.7%, and it was relatively higher for individuals who are male (22.7%), are in a younger age group (20.9%), are Coloured (33.9%), and possess medium wealth (22.5%). The prevalence of risky alcohol use was generally low (3.7%), and it is also relatively higher for those who are male (5.9%), in a younger age group (4.2%), and Coloured and White (both 4.5%), and highest for individuals with medium wealth (22.5%). More than two-thirds of the sample (68.5%) took insufficient fruits and vegetables, with more females (70%), Coloureds (73%) and African Blacks (71%), and individuals with low wealth (72.4%) and medium wealth statuses (68.5%) having higher prevalence of insufficient fruit and vegetable intake; the prevalence was evenly distributed among age groups. The prevalence of inadequate physical activity was 60.5% across the sample. This was higher among women (63.1%), the 70 and above age group (71.2%), Coloureds (76.9%), and individuals with high wealth status (62.3%). The prevalence of overweight or obesity was very high (68.2%), with females (71.9%), age group 60–69 years (71.2%), Whites (75.9%), and individuals with high wealth status having higher prevalence. Three-quarters of the sample (75.3%) had hypertension. This was higher than 70% across gender, age, race, and wealth status.

**Table 2:** Prevalence of behavioural and biological risk factors

	NCD risk factors: *N* (%)					
	
Sociodemographics	Daily tobacco use	Risky alcohol use	Insufficient fruits and vegetables	Inadequate physical activity	Overweight or obese	Hypertension
All	810 (19.7)	158 (3.7)	2,833 (68.5)	2,455 (60.5)	2,505 (68.2)	2,842 (75.3)
Gender						
Men	406 (22.7)	114 (5.9)	1,077 (62.7)	983 (57.2)	970 (63.6)	1,159 (74.4)
Women	404 (17.4)	44 (2.0)	1,534 (70.0)	1,472 (63.1)	1,535 (71.9)	1,683 (79.6)
Age						
Age 50–59	375 (20.9)	76 (4.2)	1,130 (67.7)	952 (53.2)	1,118 (68.3)	1,202 (74.9)
Age 60–69	259 (18.6)	45 (3.7)	855 (66.4)	837 (65.4)	818 (71.2)	954 (80.6)
Age 70 and above	176 (18.5)	37 (2.5)	626 (65.1)	666 (71.2)	569 (63.3)	686 (78.4)
Race						
African Black	378 (17.2)	91 (4.0)	1,478 (71.0)	1,207 (57.7)	1,338 (67.3)	1,550 (77.3)
White	58 (21.1)	7 (4.5)	144 (46.1)	169 (55.7)	190 (75.9)	193 (79.6)
Coloured	205 (33.9)	33 (4.5)	502 (73.0)	518 (76.9)	430 (71.2)	545 (85.0)
Asian or Indian	56 (18.7)	9 (1.8)	166 (52.9)	182 (52.3)	203 (67.5)	214 (76.8)
Wealth status						
Low wealth	360 (20.3)	71 (4.2)	1,074 (72.4)	931 (59.8)	817 (59.0)	1,075 (75.4)
Medium wealth	155 (22.5)	30 (4.5)	527 (68.5)	463 (58.1)	505 (72.1)	574 (78.3)
High wealth	289 (17.9)	57 (3.0)	993 (60.4)	931 (62.3)	1,170 (75.6)	1,176 (78.6)
Educational level						
No schooling	196 (21.0)	32 (2.9)	706 (71.3)	511 (59.7)	475 (67.0)	653 (76.4)
Less than 7 years	179 (21.5)	43 (4.5)	607 (69.0)	518 (59.5)	519 (70.8)	631 (78.9)
8–11 years	166 (21.7)	38 (5.3)	567 (68.6)	522 (64.4)	532 (73.3)	596 (79.2)
12 or more years	154 (17.5)	27 (3.1)	524 (60.1)	510 (59.6)	619 (81.9)	598 (75.8)
Marital status						
Single	110 (16.2)	25 (2.7)	401 (73.0)	324 (60.9)	327 (70.3)	389 (80.1)
Married or cohabiting	421 (21.3)	95 (4.4)	1,432 (64.5)	1,226 (58.2)	1,290 (71.6)	1,441 (74.2)
Separated or divorced	71 (30.2)	12 (7.3)	164 (70.7)	139 (61.8)	147 (72.8)	176 (77.7)
Widow	200 (19.1)	22 (1.7)	770 (70.9)	725 (65.4)	689 (75.2)	787 (82.9)
Geolocality						
Rural	285 (21.7)	47 (3.1)	1,036 (74.2)	780 (58.1)	748 (64.8)	916 (77.5)
Urban	523 (19.7)	111 (4.1)	1,778 (64.2)	1,673 (61.8)	1,754 (76.5)	1,923 (77.2)

Table 3 shows the calculated number of risk factors by sociodemographics. The majority of the participants (68.9%) had three or more risk factors. Table 3 shows the calculated number of risk factors by sociodemographics. The majority of the participants (68.9%) had three or more risk factors. A higher percentage of women (70.9%), individuals aged 60–69 years (84.1%), Coloured individuals (79.9%), and individuals with low (68.5%) and medium (68.9%) wealth status had three or more risk factors.

**Table 3:** Multiple risk factors classified by sociodemographics

	Number of risk factors: *N* (%)					
						Weighted mean number of NCD risk factors (95% CI)
Sociodemographics	0	1	2	3	4 or more	
All	19 (0.5)	246 (7.3)	909 (23.2)	1,343 (38.7)	1,323 (30.2)	3.01 (2.87–3.15)
Gender						
Men	10 (0.7)	142 (10.5)	408 (22.9)	437 (37.9)	523 (28.0)	3.03 (2.90–3.17)
Women	9 (0.4)	104 (4.7)	501 (23.5)	788 (39.4)	800 (32.0)	3.03 (2.90–3.17)
Age						
Age 50–59	11 (0.7)	138 (8.8)	437 (25.0)	585 (37.8)	524 (27.8)	2.89 (2.69–3.08)
Age 60–69	4 (0.4)	63 (5.4)	265 (20.0)	446 (41.6)	455 (32.5)	3.05 (2.93–3.18)
Age 70 and above	4 (0.3)	45 (6.4)	207 (23.8)	312 (36.7)	344 (32.8)	2.99 (2.81–3.17)
Race						
African Black	11 (0.5)	133 (7.1)	483 (23.0)	752 (40.0)	674 (29.4)	2.94 (2.84–3.05)
White	1 (0.9)	27 (10.8)	73 (26.7)	85 (33.6)	83 (28.0)	2.83 (2.48–3.17)
Coloured	0 (0.0)	20 (4.7)	93 (15.4)	200 (24.9)	342 (55.0)	3.45 (3.21–3.69)
Asian or Indian	1 (0.3)	25 (9.2)	95 (35.6)	119 (36.3)	67 (18.6)	2.69 (2.52–2.87)
Wealth status						
Low wealth	7 (0.4)	86 (7.1)	364 (23.1)	548 (42.9)	477 (26.6)	2.91 (2.78–3.04)
Medium wealth	1 (0.1)	50 (7.5)	149 (22.6)	252 (36.4)	279 (33.5)	3.04 (2.81–3.26)
High wealth	11 (0.9)	110 (7.5)	393 (23.7)	536 (35.9)	558 (32.1)	2.97 (2.75–3.18)
Educational level						
No schooling	3 (0.3)	45 (5.5)	204 (23.9)	318 (40.5)	284 (29.7)	2.84 (2.67–3.01)
Less than 7 years	1 (0.2)	45 (7.8)	173 (20.9)	277 (37.2)	307 (33.8)	3.01 (2.85–3.17)
8–11 years	3 (0.4)	51 (7.0)	157 (19.6)	272 (39.2)	296 (33.8)	3.04 (2.84–3.23)
12 or more years	7 (1.0)	62 (7.9)	202 (25.6)	282 (34.7)	270 (30.9)	2.87 (2.55–3.18)
Marital status						
Single	2 (0.2)	32 (7.5)	113 (23.8)	173 (39.0)	192 (29.5)	2.91 (2.78–3.04)
Married or cohabiting	12 (0.7)	159 (9.1)	513 (24.1)	682 (38.0)	641 (28.1)	3.04 (2.81–3.26)
Separated or divorced	0 (0.0)	15 (6.8)	52 (22.3)	78 (33.6)	85 (37.4)	2.97 (2.75–3.18)
Widow	4 (0.5)	37 (3.4)	210 (20.9)	386 (41.4)	383 (33.8)	3.09 (2.98–3.20)
Geolocality						
Rural	6 (0.3)	76 (6.4)	313 (22.4)	460 (43.9)	421 (26.9)	2.94 (2.77–3.10)
Urban	13 (0.6)	170 (7.7)	596 (23.7)	883 (36.0)	899 (32.0)	2.97 (2.77–3.16)

## Factors associated with multiple NCD risk factors

The mean number of NCD risk factors among all participants was 3 (95% CI: 2.81–3.10). Multivariate linear regression analysis revealed that being female, in the age group of 60–69 years and being from the Coloured and Black African population groups were associated with higher number of NCD risk factors. Marital status, educational level, wealth and residence were not significantly associated with the number of NCD risk factors experienced (Table 4).

**Table 4:** The association between multiple risk factors and sociodemographic factors

	Unadjusted		Adjusted^1^	
		
Sociodemographics	*β*	Standard error (SE)	*β*	SE
Gender				
Female	Reference		Reference	
Male	−0.17***	0.05	−0.12*	0.06
Age				
50–59	Reference		Reference	
60–69	0.17*	0.07	0.26**	0.09
70 and above	0.10	0.07	0.14	0.10
Race				
African Black	Reference		Reference	
White	−0.12	0.16	−0.12	0.13
Coloured	0.51***	0.13	0.47**	0.14
Indian or Asian	−0.25*	0.10	−0.34**	0.11
Marital status				
Single	Reference			
Married or cohabiting	−0.06	0.10	–	
Separated or divorced	0.25	0.24		
Widow	0.15	0.13		
Educational level				
No schooling	Reference		–	
Less than 7 years	0.17	0.10		
8–11 years	0.20	.09		
12 or more years	0.03	0.12		
Wealth status				
Low	Reference			
Medium	0.13	0.09	–	
High	0.06	0.12		
Geolocality				
Rural	Reference			
Urban	0.03	0.12	–	

* *p*<0.05

** *p*<0.01

*** *p*<0.001.

1 *R* ^2^=0.05

–, not available.

## Discussion

This study found that on average, elderly people in South Africa had three [95% confidence interval (CI): 2.81–3.10] NCD risk factors or unhealthy behaviours. The findings confirm the idea that unhealthy lifestyle behaviours do not happen in isolation but rather co-exist. The co-existence of unhealthy behaviours may have synergistic effects on disease risk (20).

The suffering of multiple co-existing risk factors puts elderly South Africans at an increased risk of NCDs. This may result in straining an already overburdened healthcare system. Therefore, there is a need for comprehensive and coordinated interventions within the healthcare system targeting multiple NCD risk factors that elderly South Africans face. Considering that all the NCD risk factors investigated in this study are modifiable, it signifies the urgent need for health promotion initiatives targeted at the elderly to reduce the risk for NCDs.

The study showed variations in co-existence of NCD risk factors by gender, age, and race. Women, older adults, Coloured, and Indian elderly individuals reported to have multiple NCD risk factors. Other studies also found that women had on average more NCD risk factors compared to their male counterparts (6). This calls for gender-specific interventions in addressing the risk factors for NCDs. This is in particular reference to risk factors such as physical inactivity and obesity which have been reported to be more prevalent among women than men (21, 22). Obesity among Black South African women is associated with vitality, attractiveness, physical well-being, happiness, respect, dignity, affluence, and a husband's ability to look after his wife (23–32). As in other low- and middle-income countries, contextual factors such as the absence of physical activity education, lack of safety in the neighbourhood, lack of conducive infrastructure, lower educational status, and lack of access to facilities prevent people from engaging in physical activity (26, 33). These factors must be taken into consideration when designing healthy lifestyle programmes to ensure their effectiveness. Previous studies have also reported higher numbers of NCD risk factors amongst older people (6, 34). Contextual factors, such as poverty, violence, rapid social and economic changes, lack of education, inadequate services, globalization, and urbanization, contribute to chronic NCDs in South Africa (25, 26). For example, racial differentials in the prevalence of NCD risk factors have been reported in South Africa (35). Programme developers and policy makers should address these health disparities, especially given the fact that Section 27 of the South African Constitution clearly spells out the right to health services for all citizens irrespective of race, colour, and creed. The findings of this study call for the provision of prevention, early detection, and cost-effective management of non-communicable diseases, which remains inadequate.

This study and its follow-up surveys provide a firm basis on which to monitor and evaluate health risk patterns among the elderly population in South Africa. The timing of these studies is opportune as it will inform the NHI system that South Africa is currently piloting in its quest to reduce health disparities. Monitoring health risk factors is the first step in responding to the need to reduce the burden of chronic diseases (25). However, the results of this study must be interpreted with caution as there are several limitations. First, the self-report of health variables such as tobacco or alcohol use should be interpreted with caution. It is possible that self-reports of unhealthy behaviours may be subject to social desirability biases; thus, the findings may be underestimated.

Self-reported assessments of physical activity remain the most feasible and affordable instruments for global surveillance. However, objective population measures of physical activity, such as pedometers or accelerometers (18), may be beneficial to determine whether differences between groups revealed in this study represent true differences in physical activity behaviour.

Second, this study was based on data collected in a cross-sectional survey. We cannot, therefore, ascribe causality of unhealthy behaviours to any of the associated factors in the study. Planned longitudinal studies following a cohort of the elderly will show trends and patterns of unhealthy behaviours over time. Finally, data were collected from older adults who were available in the household on the day of the survey. Respondents who were institutionalized (e.g. in a prison, hospital, or care home) and not returned to the household within 7 days and those who had moved more than 50 km away from the study household were not included.

## Conclusions

The study confirms the view that unhealthy lifestyle behaviours should not be considered in isolation from one another as they may co-exist and may also have synergistic effects on disease risk.

Thus, comprehensive health-promotion interventions not only should be tailored for specific demographic groups but also should focus on addressing multiple chronic NCD risk factors.
